# Extract from Dr. J. Allen’s Valedictory Address to the Graduates and Students of the Ohio College of Dental Surgery, at the Last Annual Commencement, February 22d, 1854

**Published:** 1854-04

**Authors:** 


*Extract from* DR. J. ALLEN'S *Valedictory Address to the Graduates and Students of the Ohio College of Dental Surgery, at the last Annual Commencement, February 22d,* 1854.

STUDENTS AND GRADUATES OF THE O. C. OF D. SURGEONS—It is with no ordinary emotion that I attempt to address you on this occasion. When I review the many interesting scenes through which we have passed during our labors here, and the harmony which has prevailed among us, I feel that ties unite us that would be hard to sever, and though time and distance may separate us—perhaps never to meet again on earth—still, while reason sits upon her throne, and memory links the past with the future, I trust we shall recall with pleasing recollection the time we have here spent together; and now as we are about to separate, permit me to say that though your assiduous efforts, thus far, have enabled you to accomplish much in the acquisition of useful theoretical knowledge, much more remains to be done. You are now in the morning of life, just entering upon the duties of your profession; you are now to carry out practically the knowledge you have acquired; you have thus far only been laying the foundation for your professional career; the superstructure has yet to be erected; the length, breadth, and hight of which will be determined by the adequate or inadequate means you employ. In the erection of a building much depends upon the style of architecture, due proportions, and decorations. The skillful architect plans his building with reference to strength, so that it shall withstand the winds and storms that may beat against it; he then adorns it with such embellishments as will make it attractive, thus combining strength with beauty; so let it be with the temple of fame that you erect for yourselves. Let the foundation be broad and deep—the superstructure magnificent and towering. Perhaps some of you may think you have not the capital with which to erect such an edifice as I have presented to your view—you will recollect, it is professional capital—a fund of knowledge that you require for this purpose, and not dollars and cents with which to employ others to build for you—professional fame—this you must do for yourselves — then it will be your own. Money often enervates the possessor of it. He that has been nursed in the lap of fortune too often inherits with his wealth effeminacy of body and mind, in consequence of which he has not the requisite force of character to ascend the hill of science, or face the stern realities of life, but is the first to quail when assailed by the rude winds of adversity; his wealth seems to render him more helpless and dependent upon others; for his independence, so far as money is concerned, will procure for him ease and luxury for the time being, without effort, and devoid of ambition, he puts forth little or no energy as a producer.

On the other hand, necessity frequently imparts strength and elasticity to the mainsprings of life. She often awakens hope, energy, and latent attributes of our nature, that might otherwise have remained dormant forever. Necessity, united with diligence and perseverance, often proves a blessing, though desired never.

" Let other nations boast of what they will, man is the nobler growth our realm supplies." Such is the genious of our government and institutions that almost any one may obtain the object of his ambition. If he desire wealth, industry, frugality and economy will procure it; if the honors of statesmen, they are within his reach; if literary distinction be his *beau ideal*, ample facilities are offered, if he will but embrace them; would he wear the wreath which genius entwines for her votaries, here are extensive resources from which he may draw freely. If you, gentlemen, desire professional excellence and distinction, you are now on the highroad that leads to it, and with perseverance you may reach the goal.

Young men are apt to think when they have completed their college course, that they know all that is important for them to learn. This conclusion is founded in error, for, in this respect, they are then only furnished with the key to the hidden mysteries of the future. If you hear a young man say he has "got through with his studies," you may regard it as synonymous with saying he has reached the zenith of his ambition in the acquisition of useful knowledge. As well might he say, on reaching the base of the Alps or the Appenines, that he had passed over the mountain-tops.

No, my young friends, if he would reach the highest pinnacle of fame, he must ascend step by step, gradually reaching one terrace after another, from each of which, as he ascends, he can look abroad, with still more extended vision, upon the expanse that widens as he nears the highest point. Many who attempt to ascend these rugged steeps fail for lack of energy. Let, then, your watchword be, onward, onward, and onward! In your advancement you will doubtless encounter many difficulties which will require your utmost capacities to surmount; hence the necessity of extensive resources in knowledge, from which to draw in time of need. But if you relax your energies, you will sink into that cold, apathetic state which characterizes the drones of society.

As professional men, you should be easy, graceful, and accomplished in your manners, with refined and well cultivated minds, combined with a thorough knowledge of all that pertains to your calling. As citizens, you should be useful members of society, ever active in carrying forward the various means that may be employed for promoting the general good of mankind. As men, be honest, just, and generous, scrupulously avoiding every loathsome habit to which so many are addicted—such as chewing tobacco, smoking, using intoxicating drinks, etc.; and, above all, avoid profanity. These, with many other pernicious practices, which, when once confirmed, are difficult to abstain from, and many, when too late, regret ever having imbibed them. Young men could easily avoid such habits, if they would—old men would, if they could.

I would recommend to you a connubial life, in preference to a life of "single blessedness." More confidence is generally reposed in a man who has a family, especially if his profession is connected with the healing art, than there is in one who leads a life of celibacy.

I would further advise you that in all cases of intricacy and doubt to move with extreme caution—examine closely, then pause and reflect—compare, and then decide. Let your decisions be firm, ever remembering that, in many instances, every hypothesis and all theories must yield to the force of circumstances.

As much will depend upon your deportment, be particularly careful never to use or tolerate any remarks which would be likely to offend the most fastidious ear.

Although cheerfulness is sometimes commendable, yet levity, in the presence of the afflicted, is never admissible.

As Christians, be fervent in spirit, relying upon him who is able and willing to do more for you than you can ever do for yourselves. In the language of Solomon: "With all thy getting, get understanding; for wisdom is the principal thing; therefore get wisdom. Exalt her, and she shall promote thee; She shall bring thee to honor, when thou dost embrace her; she shall give to thine head an ornament of grace, a crown of glory shall she deliver to thee." The wisdom here referred to pertains not only to the things of this world, but also to the world to come, which to you, of all things, is most important. Seek that wisdom which reflects light upon the path that leads to mansions in the skies.

Were it possible for you to acquire all the knowledge ever developed by science and art, and gain all the worldly honors to which you aspire, or even reach the highest pinnacle of fame, still "what shall it profit a man if he gain the whole world and lose his own soul?" Put your trust in Him who controls the destiny of man; and may you be ever blest in this life and the life to come.

*Fellow Professors:* I leave in your hands the charge of this Institution. May you continue to extend the same fostering care over it that has thus far marked its progress and prosperity. May you continue to feel the important responsibility which rests upon you. May you ever realize that, as teachers, you ingraft upon the human mind principles upon which hang, in a greater or less degree, the destiny of those over whom you preside; and they, in turn, will transmit those principles to others, in a multiplied ratio; the influence you send forth from this Institution, may sweep on, and on, and on, till time shall be no more.

